# Targeted Polymeric Nanoparticles for Brain Delivery of High Molecular Weight Molecules in Lysosomal Storage Disorders

**DOI:** 10.1371/journal.pone.0156452

**Published:** 2016-05-26

**Authors:** Marika Salvalaio, Laura Rigon, Daniela Belletti, Francesca D’Avanzo, Francesca Pederzoli, Barbara Ruozi, Oriano Marin, Maria Angela Vandelli, Flavio Forni, Maurizio Scarpa, Rosella Tomanin, Giovanni Tosi

**Affiliations:** 1 Department of Women's and Children’s Health, University of Padova, Padova, Italy; 2 Department of Life Sciences, University of Modena and Reggio Emilia, Modena, Italy; 3 Department of Biomedical Sciences, University of Padova, Padova, Italy; 4 Pediatric Research Institute “Città della Speranza”, Padova, Italy; 5 CRIBI Biotechnology Center, University of Padova, Padova, Italy; 6 Brains for Brain Foundation–Onlus, Padova, Italy; Universidad de Castilla-La Mancha, SPAIN

## Abstract

Lysosomal Storage Disorders (LSDs) are a group of metabolic syndromes, each one due to the deficit of one lysosomal enzyme. Many LSDs affect most of the organ systems and overall about 75% of the patients present neurological impairment. Enzyme Replacement Therapy, although determining some systemic clinical improvements, is ineffective on the CNS disease, due to enzymes' inability to cross the blood-brain barrier (BBB). With the aim to deliver the therapeutic enzymes across the BBB, we here assayed biodegradable and biocompatible PLGA-nanoparticles (NPs) in two murine models for LSDs, Mucopolysaccharidosis type I and II (MPS I and MPS II). PLGA-NPs were modified with a 7-aminoacid glycopeptide (g7), yet demonstrated to be able to deliver low molecular weight (MW) molecules across the BBB in rodents. We specifically investigated, for the first time, the g7-NPs ability to transfer a model drug (FITC-albumin) with a high MW, comparable to the enzymes to be delivered for LSDs brain therapy. *In vivo* experiments, conducted on wild-type mice and knockout mouse models for MPS I and II, also included a whole series of control injections to obtain a broad preliminary view of the procedure efficiency. Results clearly showed efficient BBB crossing of albumin in all injected mice, underlying the ability of NPs to deliver high MW molecules to the brain. These results encourage successful experiments with enzyme-loaded g7-NPs to deliver sufficient amounts of the drug to the brain district on LSDs, where exerting a corrective effect on the pathological phenotype.

## Introduction

Lysosomal Storage Disorders (LSDs) are a group of about 50 metabolic diseases, mostly due to the deficit of lysosomal enzymes. They are mainly characterized by a pathologic accumulation of undegraded or partially degraded substrates inside cell lysosomes and in the extracellular matrix, which affects cell and tissue functions and leads to cell death and to a general impairment of most organ-systems. LSDs are biochemically well defined and, although individually rare, they present an overall incidence of 1:8000 newborns [1]. About 75% of LSD patients present a neurological impairment, which remains mostly untreated, since recombinant corrective lysosomal enzymes, where available, cannot cross the blood-brain barrier (BBB) and reach the central nervous system (CNS) [2].

Mucopolysaccharidoses (MPSs) are a subgroup of LSDs, where the pathological storage is represented by undegraded mucopolysaccharides [3]. This study focuses in particular on the evaluation of a delivery system for brain therapeutic targeting in Mucopolysaccharidosis type I (MPS I) and type II (MPS II).MPS I (Hurler/Scheie Syndrome, MIM #607014, #607015, #607016) is an autosomic recessive disorder due to the deficit of α-L-iduronidase (IDUA, EC 3.2.1.76) [4], while MPS II (Hunter Syndrome, MIM #309900) is an X-linked recessive disease caused by the deficit of iduronate 2-sulfatase (IDS, EC3.1.6.13) [5]. In recent years, Enzyme Replacement Therapy (ERT), consisting in weekly intravenous administrations of the recombinant enzymes, has represented the most applied therapeutic approach for MPS I and II. Although systemic manifestations partly ameliorate with ERT, there is a clear evidence that the treatment has no effects on CNS disease [6]. In fact, the low level of the BBB transport system for acid hydrolases and the high molecular weight of these enzymes make any para-cellular or trans-cellular diffusion of these proteins across the BBB almost non-existent. Therefore, efficient methods to achieve a safe transcytosis into the CNS need to be explored.

Invasive techniques for BBB crossing have been based on neurosurgery or on a temporary chemical/physical disruption of the barrier, produced by biochemical and immunological changes or by an osmotic shift [7–9]. However, both these approaches entail several drawbacks such as the invasiveness and the high costs of neurosurgery, the physiological stress or the transient increase of the intracranial pressure, along with high risk of infections and damages from toxins, due to the BBB temporary opening. Therefore, to improve drug delivery to the brain, non-invasive techniques have been explored and, among them, the nanotechnology-based approach surely represents one of the most encouraging.

Polymeric nanoparticles (NPs) have shown to be promising carriers for CNS drug delivery, due to their potential both in encapsulating drugs, hence protecting them from the body excretion and metabolism, and in delivering therapeutic molecules across the BBB without inflicting any damages to the barrier itself [10,11]. Different polymers were employed to design NPs, poly(butyl cyanoacrylate) (PBCA) or poly(isohexyl cyanoacrylate) (PIHCA), poly(lactic acid) (PLA) or its copolymer poly(lactide-co-glycolide) (PLGA), human serum albumin (HSA) and chitosans are considered the most promising materials in reason of their performances and properties as biodegradability and biocompatibility or suitability in drug formulation [12]. In fact, these systems are able to efficiently encapsulate a variety of molecules as anticancer drugs or other small therapeutic molecules, through the BBB [13]. Among them, mainly the use of poly-lactide-co-glycolide (PLGA), an FDA-approved polymer, together with specific ligands, rendering the delivery of drugs to CNS more targeted, has been more recently considered [14–16]. In details, PLGA-NPs derivatized with the peptide H_2_N-Gly-*L*-Phe-*D*-Thr-Gly-*L*-Phe-*L*-Leu-*L*-Ser(O-β-*D*-Glucose)-CONH_2_ [g7] are able to efficiently cross BBB [17] without damaging [18]. Moreover, the fate of g7-NPs was investigated both in cultured neuronal cells and in mice, thus elucidating the uptake pathways in neurons. In particular, clathrin and Rab-5 pathways were identified as responsible for NPs uptake and trafficking into neuronal cells.

Interestingly, following intracellular passage, g7-NPs were found to be transferred, in different percentages, from cell to cell by tunneling nanotubes, released outside the cells or accumulated in lysosome vesicles [14]. This last finding, which commonly represents a drawback in drug delivery to neuron cytoplasm, as recently reported [19], in the case of LSDs can be considered, on the opposite, an advantage since lysosomes, site of NPs accumulation, are themselves the targets of LSDs therapy.

At present, some clinical trials and preclinical evaluations proposed the use of PLGA-NPs as drug carriers in cancer therapy [20–23], but so far none evaluated their use for the treatment of CNS diseases. In the context of LSDs, the set-up of a therapeutic protocol successfully addressing the neurological impairment would dramatically improve patients’ conditions.

Before going into efficacy studies, we here developed several preliminary experiments aimed to explore, for the first time, the ability of g7-NPs to efficiently load and transfer across the BBB a high molecular weight molecule, using a model drug (FITC-albumin). Experiments, conducted on both MPS I and MPS II mouse models, included a whole series of control administrations, allowing a broad preliminary view.

## Materials and Methods

### Animal models

The C57BL/6 Idua knockout (Idua-ko) mouse, providing the model for MPS I, generated in the laboratory of Dr. Neufeld (David Geffen School of Medicine, UCLA, USA), was kindly provided by Dr. Heard (Pasteur Institute, Paris, France); the mouse was generated by targeted gene disruption of the murine Idua gene and previously characterized [24].

The C57BL/6 Ids knockout (Ids-ko) mouse, providing the model for MPS II, was a kind gift of Dr. Muenzer (University of North Carolina, NC, USA); it was generated by gene disruption of the murine Ids gene and it has been previously characterized [25–27].

Both colonies were expanded in our animal facility and mice were genotyped by multiplex PCR. In the experiments here described Idua-ko, Ids-ko and wild type (wt) mice were housed in light and temperature controlled conditions, with food and water provided ad libitum. This study was carried out in strict accordance with the recommendations in the Guide for the Care and Use of Laboratory Animals of the National Institutes of Health. The protocol was approved by the Ethical Committee for Animal Experimentation of the University of Padova (Permit Number: 2/2013).

### Chemicals

Poly(D,L-lactide-co-glycolide) (PLGA,RG503H, MW near 11,000) was used as received from the manufacturer (Boehringer-Ingelheim, Ingelheim am Rhein, Germany). Polyvinyl alcohol (PVA, MW 15,000) was purchased from Sigma-Aldrich (Milan, Italy). Gly-*L*-Phe-*D*-Thr-Gly-*L*-Phe-*L*-Leu-*L*-Ser(O-β-*D*-Glucose)-CONH2 (g7) linked to biotin was synthesized as previously reported and purchased from Mimotopes (Clayton, Victoria, Australia) [14]. PLGA conjugated with Rhodamine B piperazine amide (Sigma-Aldrich) (R-PLGA) was prepared as previously described [28]. Albumin-fluorescein isothiocyanate conjugate (FITC-Albumin) was purchased from Sigma-Aldrich (A9771). Maleimide activated Neutravidin (NA-Maleido) was obtained by Thermo Scientific. A MilliQ water system (Millipore, Bedford, MA, USA), supplied with distilled water, provided high-purity water (18 MΩ). All other chemicals were of analytical grade.

### Preparation of NPs

Nanoparticles (NPs) were prepared by a double emulsion technique [29,30]. Briefly, 1 mL of FITC-albumin water solution (20mg/ml) was emulsified in 5 mL CH_2_Cl_2_ solution of polymer (95 mg of PLGA and 5 mg of R-PLGA) under cooling (5°C) by using a probe sonicator (MicrosonUltrasonic cell disruptor, Misonix Inc. Farmingdale, NY, USA) at 80W for 45 sec to obtain a w/o emulsion (first inner emulsion). The first inner emulsion was rapidly added to 12 mL of 1% (w:v) PVA aqueous solution and the w/o/w emulsion was formed under sonication (80W for 45 sec) at 5°C. Formulation was mechanically stirred (1,500 rpm) for at least 1 h (RW20DZM, Janke&Kunkel, IKA-Labortechnik, Staufen, Germany) at RT until the complete solvent evaporation and finally purified by Hi-Speed Refrigerated Centrifugation (Beckman J21) at 17,000 rpm for 10 min at 5°C, washed several times with water and re-suspended in water. This technique allowed to obtain labeled Alb-loaded un-targeted NPs, used as control. Also, un-loaded NPs were obtained by using a saline solution (150 mM NaCl) instead of the FITC-albumin solution, leading to un-targeted un-loaded NPs (u-NPs), used as control as well.

To obtain brain targeted NPs (un-loaded g7-NPs or loaded g7-NPs/Alb) g7 was conjugated on the NPs surface using a post-modification approach [31]. Briefly, 50 mg of each preparation was suspended in 2-(N-morpholino) ethanesulfonic acid (MES, Sigma Aldrich) buffer and reacted with 50 mg of N-Hydroxy-succinimide (NHS, Sigma Aldrich) plus 150 mg of 1-Ethyl-3-(3-dimethylaminopropyl)-carbodiimide (EDC; Sigma Aldrich, Saint Luis, MO) to activate carboxylic group of the polymer. After 1 hour, activated NPs were collected by ultracentrifugation at 17,000 rpm for 10 min at 4°C and the excess of reagents was removed. NPs were re-suspended in PBS pH 7.4 and reacted with 2 mg neutravidin(NA)-Maleido (Sigma- Aldrich) previously activated by adding 20 μl of cysteamine (2mg/ml). Mixture was mechanically stirred for 2 hours at RT and the neutravidin-modified NPs (NA-u-NPs or NA-u-NPs/Alb) were purified by Hi-Speed Refrigerated Centrifugation (Beckman J21) at 17,000 rpm for 10 min at 5°C. Finally, biotinylated g7 peptide (2.6 mg) was solubilized in 1 ml of water and reacted with NA-NPs for 1 hour at room temperature. g7-NPs were than purified by centrifugation as previously described for un-targeted NPs.

All NP formulations were frozen using trehalose as cryoprotectant [32] (1:1 w:w polymer/trehalose ratio) and stored at -20°C until use. In all batches the percentage of R-PLGA is maintained constant allowing a comparison of the NPs sample in terms of fluorescence.

Notably, by this procedure we were able to obtain NPs loaded with albumin inside (u-NPs/Alb and g7-NPs/Alb) and by the purification protocols, we were able to reduce up to not-significant levels the Alb which remains adsorbed onto NPs surface.

Other control samples included a mixture of un-loaded targeted NPs (g7-NPs) suspended in the FITC-albumin solution (MIX1) and un-loaded un-targeted NPs (u-NPs) suspended in the FITC-albumin solution (MIX2).

**Fig 1:**
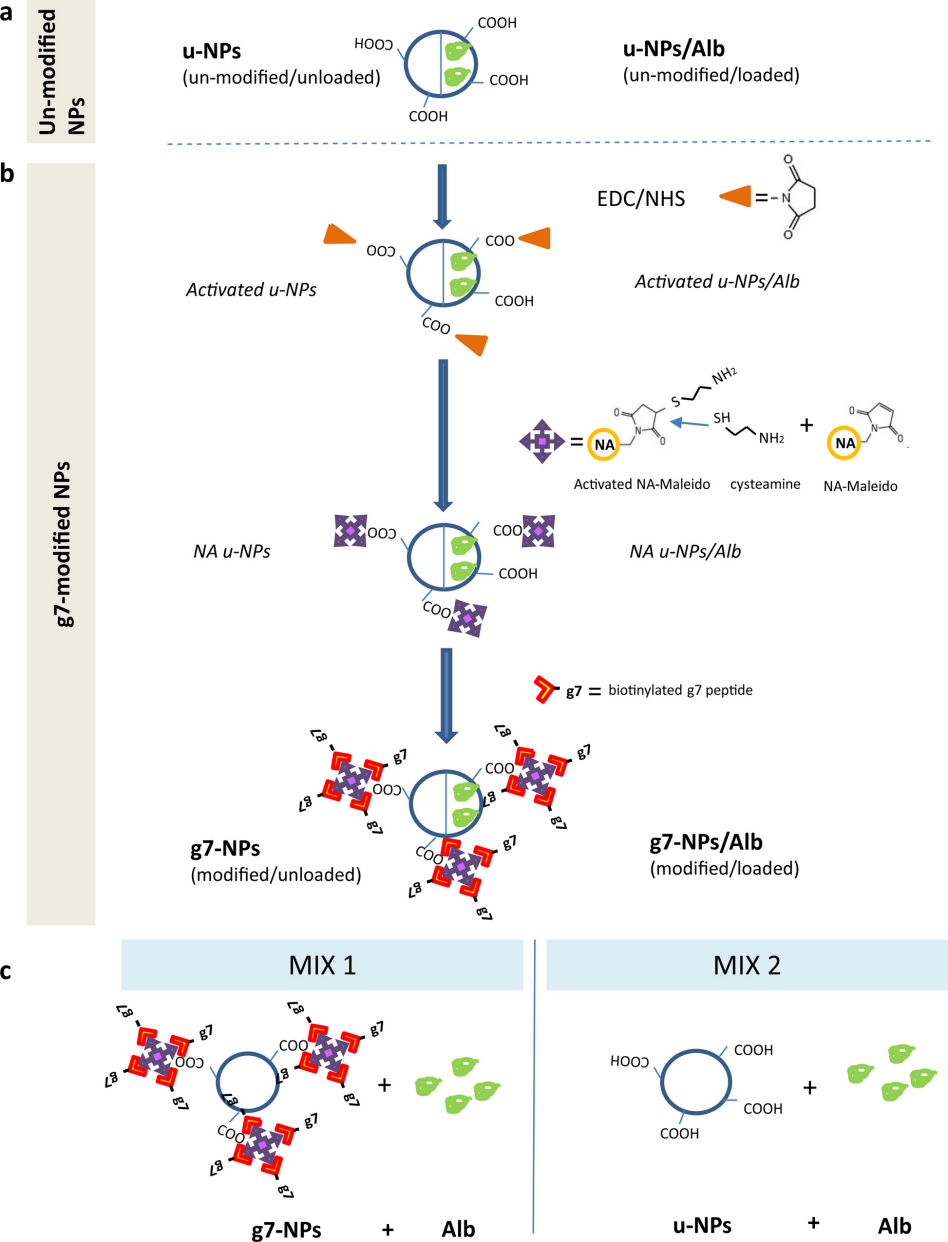
Preparation scheme of NP samples. (a) Un-modified NPs were prepared both as un-loaded NPs (empty left half circle) and as albumin loaded-NPs (right half circle). b) Un-modified NPs were chemically modified on their surface (EDC/NHS activation followed by NA-maleido reaction and biotinylated-g7 conjugation) in order to produce modified NPs both un-loaded (empty left half circle) and loaded with albumin (right half circle). c) MIX samples: MIX1 = physical mixture of un-loaded g7-NPs + albumin solution; MIX2 = physical mixture of un-loaded u-NPs + albumin solution. u-NPs = un-modified NPs, Alb = albumin, EDC = 1-Ethyl-3-(3-dimethylaminopropyl)-carbodiimide, NHS = N-hydroxysulfoxuccinimide, NA = neutravidin.

A summary of all NPs preparation is represented in Fig 1.

#### Chemico-physical NPs characterization

All batches of NPs were characterized for surface, chemico-physical and morphological properties. In particular, for surface properties (size and charge), NPs in 150 mM NaCl were analyzed by photon correlation spectroscopy (PCS) and laser Doppler anemometry using a Zetasizer Nano ZS (Malvern, UK; Laser 4 mW He–Ne, 633 nm, Laser attenuator Automatic, transmission 100% to 0.0003%, Detector Avalanche photodiode, Q.E. > 50% at 633 nm, T = 25°C). Results were normalized with respect to a polystyrene standard solution.

To evaluate shape and morphology of NPs, a scanning electron microscope (SEM) (XL-40; Philips, Eindhoven, The Netherlands) operating at 8 kV was used. Following at least 3 washings, NPs were re-suspended in distilled water and a drop of the suspension was placed onto a SEM sample holder and dried under vacuum (10–2 mmHg). Dried samples were coated under argon atmosphere with a 10 nm gold palladium thickness (Emitech K550 Super Coated; Emitech Ltd, Ashford, Kent, UK) to increase electrical conductivity. The NPs were then processed for the evaluation of their morphology and shape, performed by analyzing images at different magnification (13,000× to 16,000×). Also Atomic Force Microscopy (AFM) was used for morphological assessments. In particular, AFM was used (Park Instruments, Sunnyvale, CA, USA) at RT (about 20°C), at atmospheric pressure (760 mmHg) operating in air and in non-contact mode, using a commercial silicon tip-cantilever (tip diameter ≈ 5–10 nm) with stiffness about 40 Nm-1 and a resonance frequency around 150 kHz. A little amount of each NP sample was dispersed in deionized water (about 40 μL) on a small freshly cleaved mica disk (1 cm x 1 cm). Two minutes after the deposition, the excess of deionized water was removed by a blotting paper and the sample observed. Two kinds of images were obtained: a topographical image and the 3D elaboration. The AFM images were obtained with a scan rate of 1 Hz and processed using a ProScan Data Acquisition software developed under Windows 95.

#### Albumin content

Freeze-dried NPs (5 mg) were dissolved in 1 mL of dichloromethane. Then, 3 mL of PBS pH 7.4 were added to extract the FITC-albumin and the organic solvent was evaporated at RT under stirring (1,500 rpm for at least 1 h; RW20DZM, Janke&Kunkel, IKA-Labortechnik, Staufen, Germany). The aqueous solution was filtered (cellulose acetate filter, porosity 0.2 μm, Sartorius) to remove the polymer residues and spectrophotometrically analyzed at 492 nm to evaluate the FITC-albumin concentration. Drug loading was expressed as mg of FITC-albumin encapsulated/100 mg of NPs and as encapsulation efficiency (EE%), i.e. the percentage of encapsulated drug related to the initial amount of drug used in the preparation.

#### ESCA analyses

The presence of g7 on NPs surface was demonstrated by electron spectroscopy for chemical analysis (ESCA), showing the presence of nitrogen atoms on the surface of antibody-engineered NPs, as previously described [33]. ESCA was performed on a XRC 1000 X-ray source analysis system (Specs Surface Nano Analysis, Germany) and a Phoibos 150 hemispherical electron analyzer (Specs Surface Nano Analysis, Germany), using MgKα1,2 radiations. Spectra were recorded in fixed retardation ratio (FAT) mode with 40 eV pass energy. The pressure in the sample analysis chamber was around 10−9 mbar. Data were acquired and processed using the SpecsLab2 software.

### Animal procedures and tissue samples preparation

Idua-ko, Ids-ko and matched wt mice (5–8 months old) were intravenously (i.v.) injected as indicated in Table 1. For each batch of PLGA-NPs, 2 mg NPs were intravenously injected in each mouse; as for albumin, 0.2 mg/animal were injected, either free or NPs-loaded.

**Table 1:** Type of injection performed in the two mouse models.

*MOUSE MODEL*	*TYPE OF INJECTION*	*NUMBER OF MICE*
*Idua-ko/wt*	g7-NPs	3
	u-NPs	3
	g7-NPs/Alb	3
	u-NPs/Alb	3
	MIX1 (g7-NPs+Alb)	3
	MIX2 (u-NPs+Alb)	4
	Alb	3
	EB	3
*Ids-ko/wt*	g7-NPs/Alb	5
	Alb	3
	EB	3

Idua-ko: α-L-iduronidase knock-out mice; Ids-ko: iduronate 2-sulfatase knock-out mice; wt: syngeneic wild-type mice; g7-NPs: un-loaded targeted nanoparticles; u-NPs: un-loaded un-targeted nanoparticles; g7-NPs/Alb: targeted nanoparticles loaded with albumin; u-NPs/Alb: un-targeted nanoparticles loaded with albumin; MIX1 (g7-NPs+Alb): un-loaded targeted nanoparticles suspended in FITC-albumin solution; MIX2 (u-NPs+Alb): un-loaded un-targeted nanoparticles suspended in FITC-albumin solution; Alb: Albumin; EB: Evans Blue solution.

3–5 animals were sacrificed 2 h post-injection by cervical dislocation for all type of treatment. This time interval was chosen as being sufficient to allow an efficient BBB crossing by the NPs, but also representing a time-point at which the release of Alb from NPs could be considered sufficiently low. Therefore we can truly consider that almost all Alb loaded into NPs is still stably encapsulated. Mouse tissues (brain and liver) were collected and fixed in 4% paraformaldehyde and 0.2% picric acid in 1X PBS for 48 h. Afterwards, tissues were subjected to a sucrose gradient: 15% sucrose in PBS for 24 h and then 30% sucrose in PBS for additional 24 h. Finally, tissues were embedded in Tissue-Tek® O.C.T™ Compound (Sakura Finetek Europe, Leiden, NL) and snap-frozen using dry ice. Before freezing, brains were cut at bregma level. Serial 5 μm thick cryosections, from the bregma to the cerebellum, were obtained, washed 3 times in cold PBS, mounted with Fluoromount™ (Sigma-Aldrich) with DAPI (BioChemica, AppliChem GmbH, Darmstadt, Germany) and stored at +4°C until analysis.

### Confocal analysis

Confocal analysis (Confocal Microscope Leica DM IRE 2, Bannockburn, IL, USA) was performed following a procedure previously reported [34]; all samples were analyzed in triplicate using the Fiji distribution of ImageJ software [35]. In particular, the evaluation of hippocampus, cortex and dentate gyrus on confocal microscopy was performed by counting at least 6–8 optical fields (with 20 as average number of nuclei in each field) per slice. The values represent the average of at least 5 slices per each brain.

### Statistical analysis

Statistical evaluation was performed by using Student’s t-test. Differences were considered statistically significant for p < 0.05.

## Results and Discussion

Drug delivery to the brain represents one of the most stimulating challenges in neuro-research, giving the limited number of therapeutics allowed to reach the CNS. In fact, 98% of the drugs are unable to cross the BBB owing to their high molecular weight or chemico-physical properties [36]. To improve drug delivery to the brain, non-invasive techniques have been investigated and, among them, the nanotechnology-based approach surely represents one of the most promising. With the aim to move into functional and efficacy studies, targeted to treatment of the brain disease in Lysosomal Storage Disorders, we here conducted, for the first time, several preliminary experiments in the murine models of MPS I and MPS II to explore the ability of g7-NPs to deliver across BBB a model drug (FITC-albumin), with a high molecular weight, similar to the therapeutic enzymes commonly used in enzyme replacement therapy protocols.

### NPs features

All NPs were characterized in terms of size, surface charge, drug loading and surface composition (Table 2). In particular, all NPs, independently from surface modification or loading, were featured by size under 300 nm, thus compatible with a systemic administration, good homogeneity (as PDI values are ranging between 0.08 and 0.1 indicating an homogeneous population of NPs) and negative surface charges, as usual when considering PLGA NPs [17].

**Table 2:** Chemico-physical characterization of nanoparticles.

Samples	Medium	Z-Average nm (±S.D)	PDI^a^ (±S.D)	Di50^a^ nm (±S.D)	Di90^a^ nm (±S.D)	ζ-pota mV (±S.D)	mg of FITC Alb/100 mg NPs (±S.D)	EE%^b^ (±S.D)	N/C ratio
**u-NPs**	**DI water**	221 (7)	0.08 (0.02)	230 (9)	330 (4)	-16 (4)	/	/	0.010
	**NaCl 150 mM**	216 (6)	0.08 (0.04)	228 (7)	325 (6)	-11 (5)	/	/	0.010
**g7-NPs**	**DI water**	234 (8)	0.11 (0.03)	231 (5)	335 (9)	-14 (3)	/	/	0.024
	**NaCl 150 mM**	228 (5)	0.12 (0.04)	231 (5)	329 (6)	-9 (4)	/	/	0.024
**u-NPs/Alb**	**DI water**	260 (7)	0.11 (0.03)	275 (5)	480 (8)	-21 (5)	10.4 (1.1)	50 (5)	0.012
	**NaCl 150 mM**	254 (5)	0.10 (0.05)	271 (4)	472 (4)	-15 (5)	10.4 (1.1)	50 (5)	0.012
**g7-NPs/Alb**	**DI water**	261 (8)	0.08 (0.02)	268 (4)	375 (9)	-24 (4)	7.3 (0.1)	36 (4)	0.025
	**NaCl 150 mM**	254 (4)	0.08 (0.02)	263 (4)	371 (6)	-17 (3)	7.3 (0.1)	36 (4)	0.025

N/C is reported for each NPs sample; as commonly accepted, when the molecule (i.e. g7 peptide) modifying the surface of NPs is rich of N, the presence of N signals onto NPs surface is considered as the proof of the surface engineering. The lower is the N/C value the lower is the presence of the molecule modifying the NPs surface. Di50 and Di90 are referring to dimensional distribution values (50 and 90).

^a^ Values are given as mean±S.D. (n = 9).

^b^ The percentage of encapsulation efficiency was determined as the ratio of the encapsulated out of the total (encapsulated + free) drug per cent (%). Values are intended as mean±S.D. (n = 9).

u-NPs: un-loaded un-targeted nanoparticles;g7-NPs: un-loaded targeted nanoparticles; u-NPs/Alb: un-targeted nanoparticles loaded with albumin;g7-NPs/Alb: targeted nanoparticles loaded with albumin; DI water: deionized water; Z-Average: average diameter of nanoparticles; PDI: Poly dispersity index; ζ-pot: zeta potential indicating surface charge; EE: encapsulation efficiency; N/C: C = percentage of Carbon signals onto NPs surface and N = Nitrogen signals; Di50 and Di90: dimensional distribution values (50 and 90).

Some differences in chemico-physical parameters could be recognized by comparing unloaded and loaded NPs; in particular, loaded samples, formulated at 1:5 drug to polymer molar ratio, were characterized by a significant increase in Z-Average diameter (form 230 to 260), probably due to the encapsulation of a high MW molecule, but resulting however in an homogeneous size distribution (PDI = 0.01). In these samples, ζ-pot slightly decreased (about -22 mV) probably for the different reorganization of polymer chains in the presence of loaded albumin.

Percent of loading efficiency (EE%) of u-NPs and g7-NPs is quite different, with a decrease in Alb content of about 30% in g7-NPs/Alb with respect to u-NPs/Alb. This data is not surprising, since the loading process with Alb happens as first step in the formulation of NPs, followed by activation of NPs and conjugation with peptides, suspended and stirred in aqueous medium, thus leading to a possible loss in drug content [37].

Interestingly, the surface analysis of NPs by ESCA described the presence of g7 onto NPs surface, as confirmed by an increased nitrogen/oxygen ratio in g7-NPs (both unloaded and loaded with Alb). The same data were not recovered for un-modified NPs and nicely correlated with previous reports on the surface engineering of PLGA NPs [37–40]. The analysis conducted on the activated NPs (intermediate step of formulation obtained by neutravidin activation) did not show a significant increase in N/C ratio with respect to un-modified NPs (i.e. N/C ratio = 0.12) (data not shown) as previously reported [37–40]. This confirmed that the amount of nitrogen onto activated NPs is negligible with respect to that derived from g7 surface modification.

**Fig 2:**
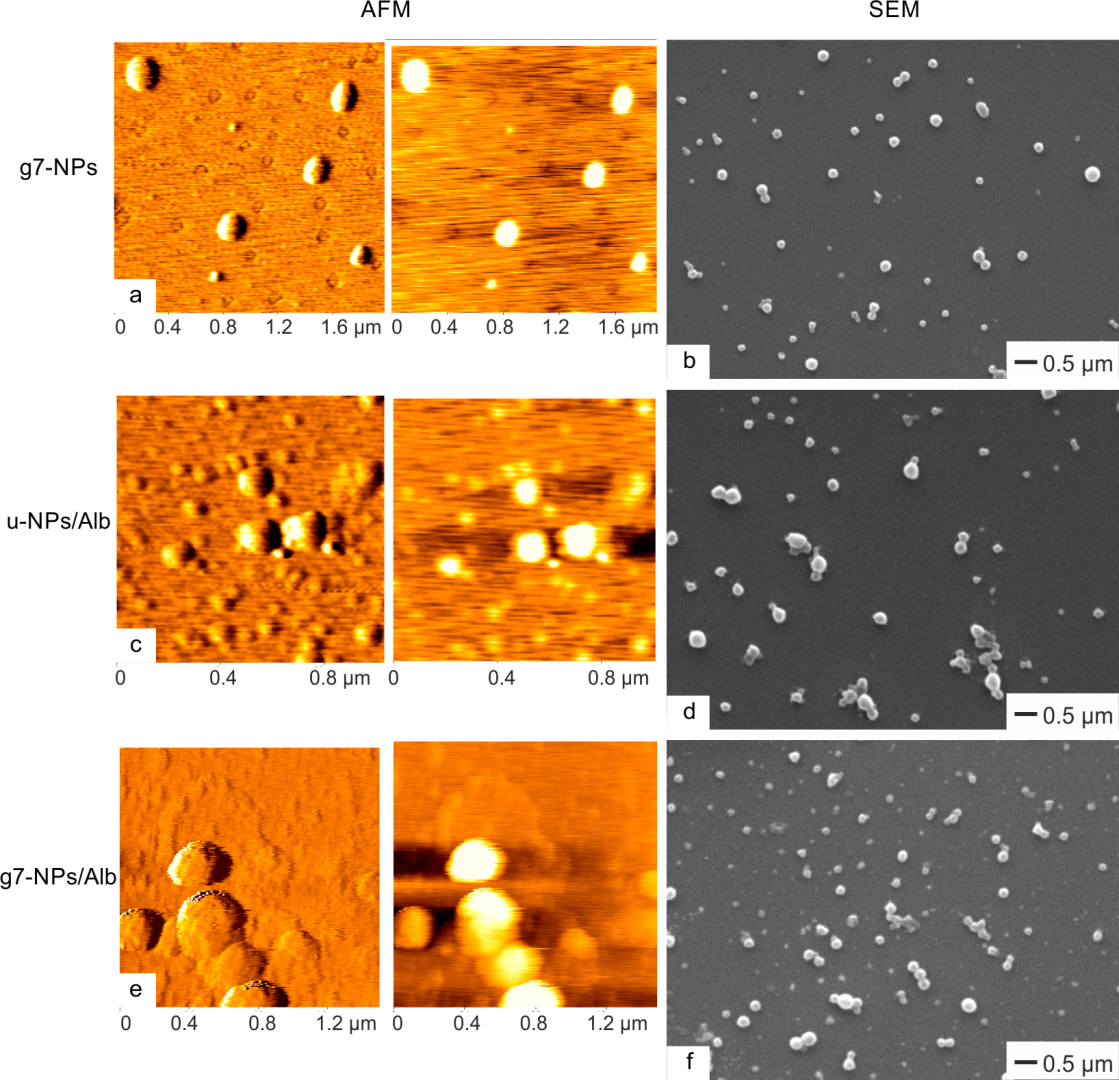
Morphologic characterization of nanoparticles. AFM (panels a, c, e) and SEM (panels b, d, f) images of unloaded g7-NPs, used as control, loaded u-NPs/Alb and g7-NPs/Alb, respectively. g7-NPs: unloaded targeted nanoparticles; u-NPs/Alb: untargeted nanoparticles loaded with albumin;g7-NPs/Alb: targeted nanoparticles loaded with albumin.

From a morphological point of view, AFM and SEM (Fig 2A and 2B) analyses confirmed that unloaded samples were formed by spherical structures with a diameter in agreement with PCS data and regular surface.

Considering loaded NPs, microscopic examination, and particularly AFM outputs, displayed more complicated structures. Although still spherical, u-NPs/Alb showed a discontinuous and rough surface (Fig 2C and 2D). On the opposite, g7-NPs/Alb appeared with a more regular and homogeneous surface; this fact is likely to be attributed to the surface modification process, when the adsorbed FITC-albumin is partially removed and washed away from the NPs surface. This finding is also evident by the content analysis previously exposed, evidencing a decrease of Alb content in g7-NPs versus u-NPs. Moreover, a slight tendency to aggregation, once on mica surface, could be highlighted in the case of g7-NPs, probably due to the presence of neutravidin and g7 on the surface (Fig 2E and 2F).

### Study of PLGA-NPs and albumin BBB crossing in the MPS I mouse model

**Fig 3:**
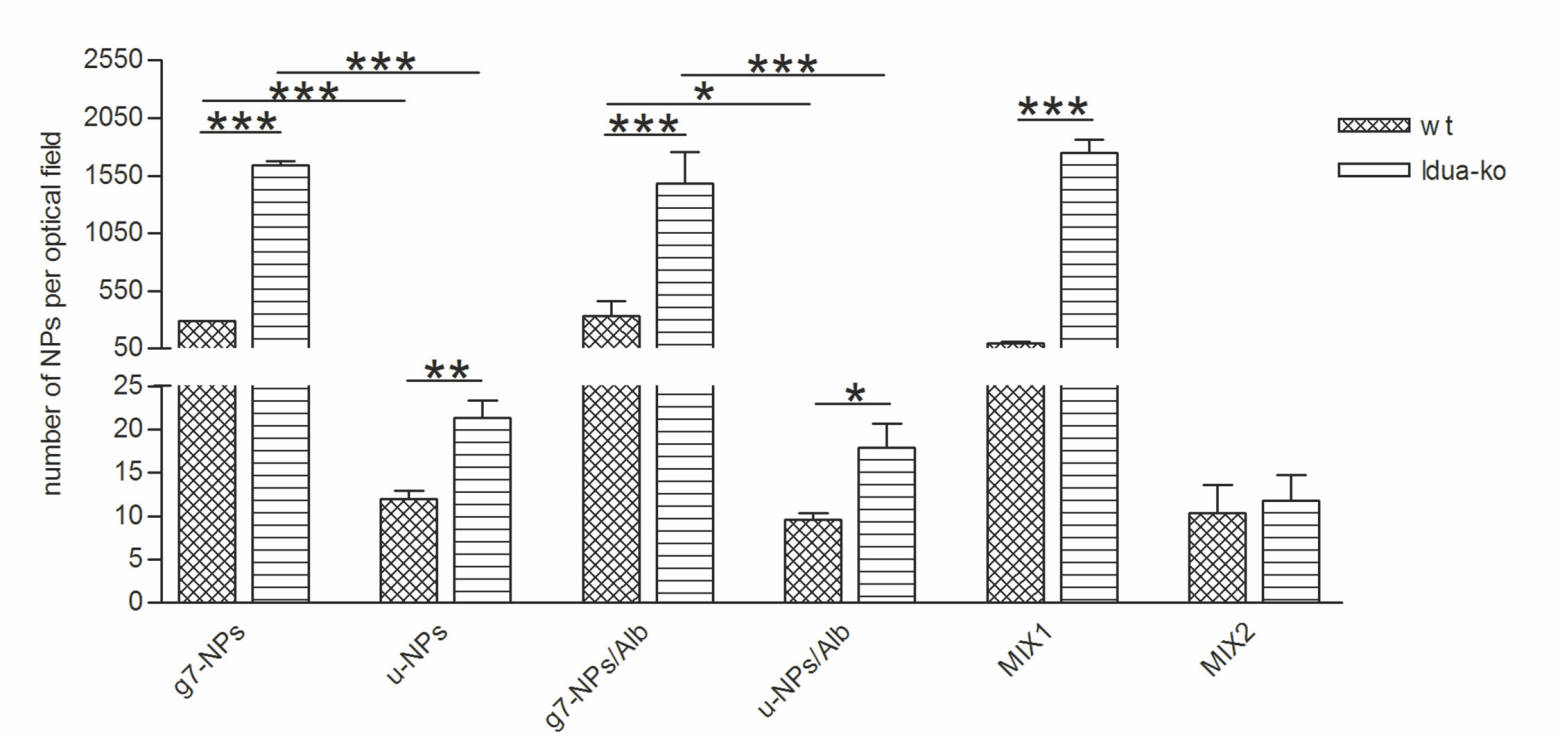
Number of NPs in the brain of Idua-ko and wt mice. g7-NPs: unloaded targeted nanoparticles; u-NPs: unloaded untargeted nanoparticles; g7-NPs/Alb: targeted nanoparticles loaded with albumin: u-NPs/Alb, untargeted nanoparticles loaded with albumin; MIX1 (g7-NPs+Alb): unloaded targeted nanoparticles suspended in FITC-albumin solution; MIX2 (u-NPs+Alb): unloaded untargeted nanoparticles suspended in FITC-albumin solution. *p-value <0.05.

Baseline experiments, aimed to confirm in the MPS I animal model results previously obtained by our group in the wt mouse, included injection of 2 mg/animal of g7-NPs, or u-NPs, in the tail vein of Idua-ko and wt mice (n = 3 for each treatment and each type of mouse) [14–17]. As shown in Fig 3 and image in S1 Fig, results confirmed for the Idua-ko mouse model, and for the syngeneic wt control, an efficient g7-NPs crossing of the BBB, with a higher efficiency, of about 5.5 fold, for the Idua-ko (mean value: 1641 g7-NPs per optical field) vs the wt mouse (mean value: 288 g7-NPs per optical field). On the opposite, u-NPs BBB crossing looked extremely poor or totally absent in our wt mouse (mean value: 12 u-NPs per optical field), as previously shown in other rodents [17], and slightly higher in the Idua-ko mouse, although still very low (mean value: 21 u-NPs per optical field).

BBB status in LSDs was previously investigated. In particular, evaluations performed in the animal models of Gaucher disease and GM1 gangliosidosis showed important BBB alterations, while analysis of the barrier in the mouse model of the late onset Tay-Sachs disease did not reveal any abnormalities [41,42]. In addition, in the MPS III condition, endothelial cell damage with possible compromise of the BBB was described, indicated, among others, by Evans Blue and albumin microvascular leakage, in various brain structures [38,39,43]. On the opposite, a similar analysis conducted in this work for both wt and Idua-ko mice, by using Evans Blue (S1 Text) and FITC-albumin, revealed no altered permeability, thus possibly suggesting modified pathways of uptake.

**Fig 4:**
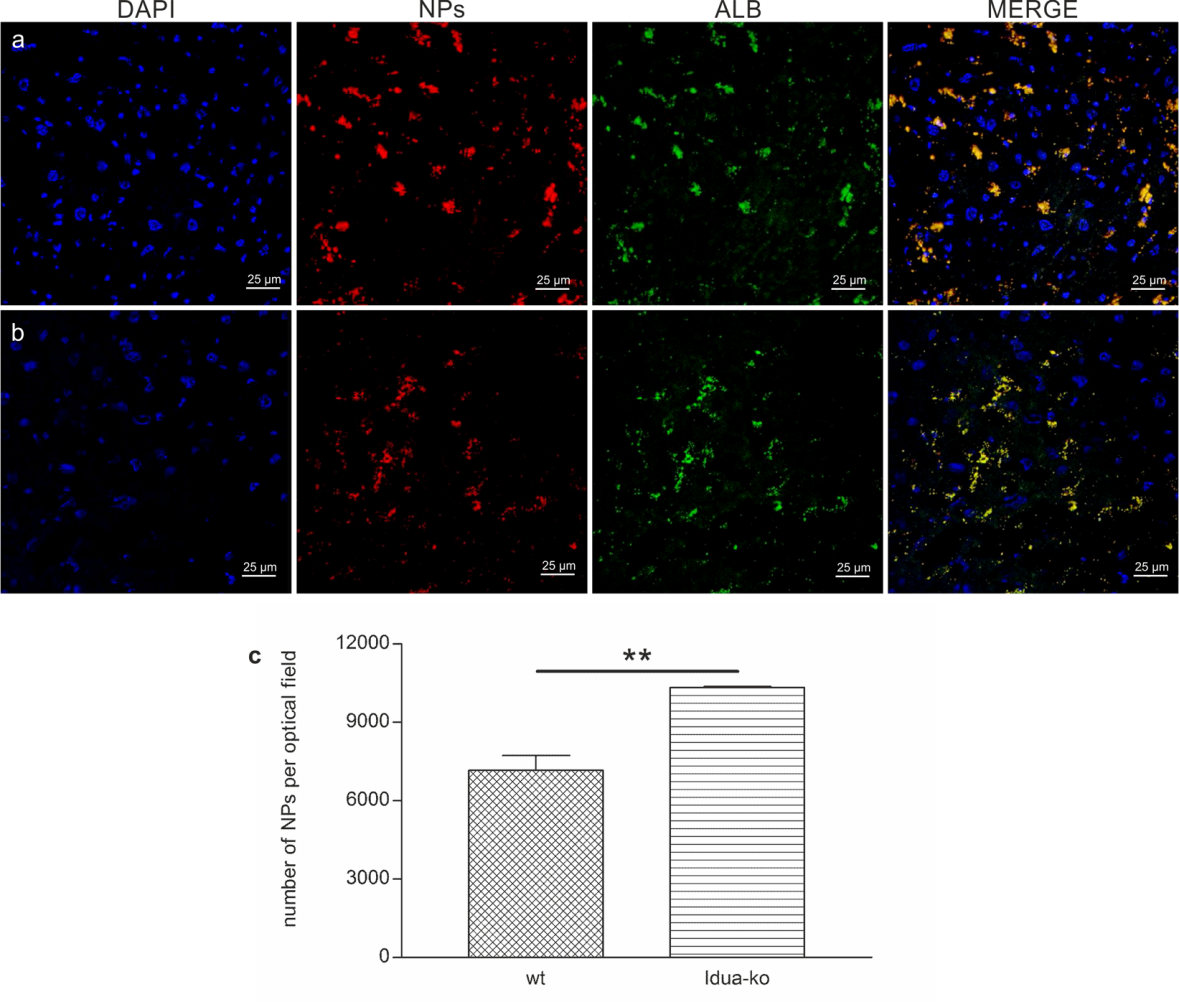
Number of g7-NPs/Alb and representative confocal images of Idua-ko and wt mice liver. (a) Idua-ko and (b) wt mice. Nuclei are shown in blue (DAPI staining), NPs in red (rhodamine labeling), Albumin in green (FITC labeling); last column represents the merged images. (c) Number of g7-NPs/Alb per optical field. **p-value <0.01.

This hypothesis is supported by the fact that also in the liver a higher efficiency of uptake in the Idua-ko vs the wt mouse was found (Fig 4), suggesting also for other organs that altered uptake pathways may be implicated. This way, NPs, although with remarkable advantages in terms of success (g7-NPs˃˃u-NPs), could exploit the improved transcytotic pathways to reach Idua-ko brains, similarly to what previously reported with the brain targeted pro-drug of the α-L-iduronidase therapeutic enzyme (with receptor binding peptide for ApoE3) [40].

**Fig 5:**
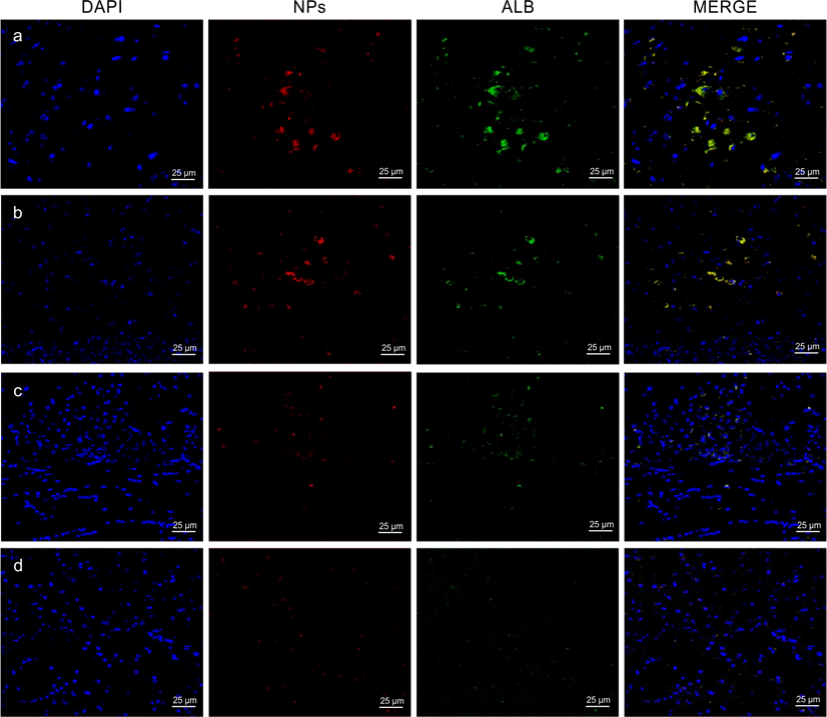
g7-NPs/Alb and u-NPs/Alb representative confocal images of Idua-ko and wt mice brain. (a), (c) Idua-ko mice; (b), (d) wt mice; (a), (b) g7-NPs/Alb; (c), (d) u-NPs/Alb treatments. Nuclei are shown in blue (DAPI staining), NPs in red (rhodamine labeling), Albumin in green (FITC labeling); last column represents the merged images. g7-NPs/Alb: targeted nanoparticles loaded with albumin; u-NPs/Alb: untargeted nanoparticles loaded with albumin.

**Fig 6:**
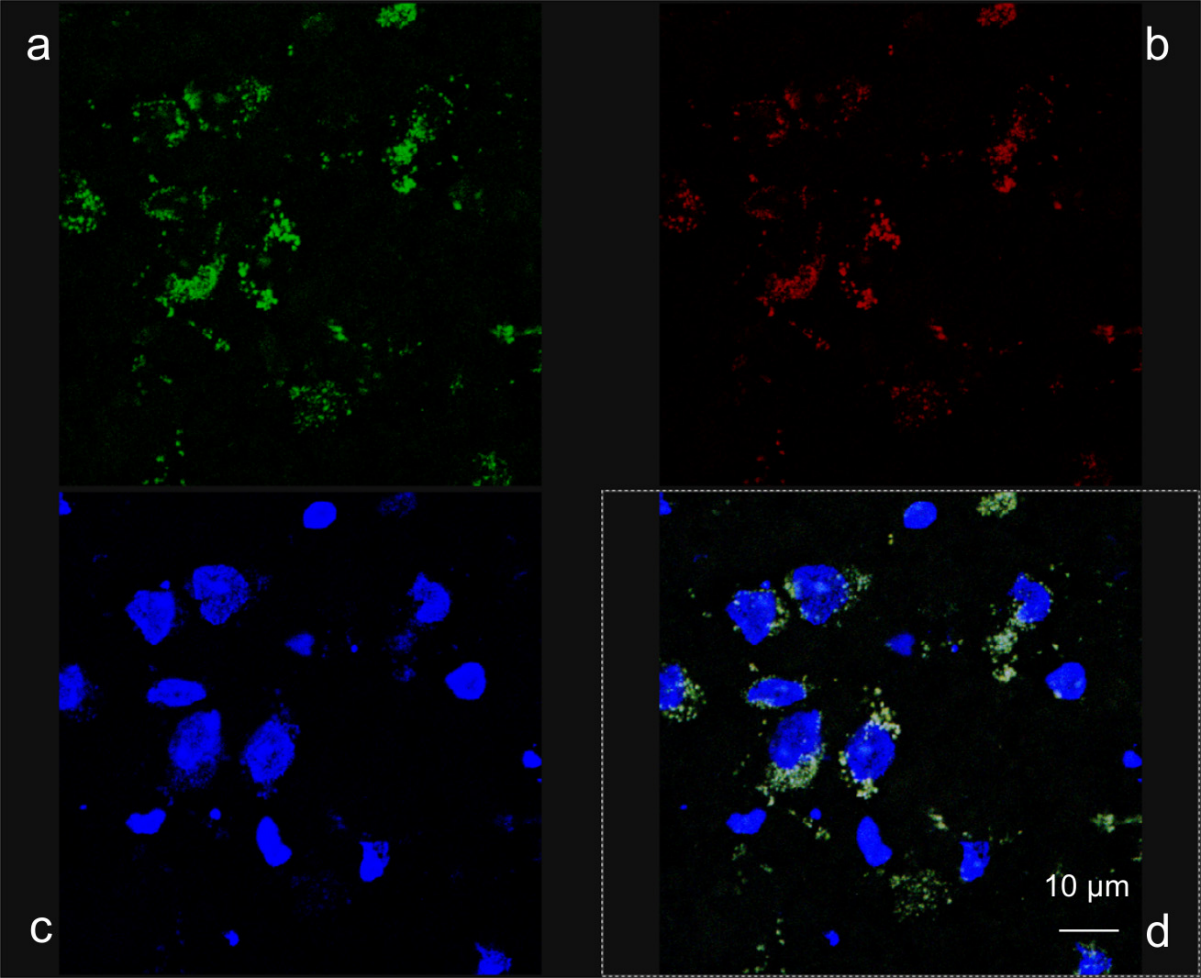
Localization of g7-NPs/Alb in Idua-ko mouse brain. Representative confocal images of the g7-NPs/Alb perinuclear localization in the brain of Idua-ko mice injected with g7-NPs/Alb. (a) Albumin is shown in green (FITC labeling), (b) NPs in red (rhodamine labeling), (c) nuclei in blue (DAPI staining); (d) represents the merged images.

We next administered targeted nanoparticles loaded with albumin (g7-NPs/Alb) in both animal types (Idua-ko n = 3, wt n = 4) to evaluate efficiency of g7-NPs BBB crossing as well as ability to carry over the albumin. As shown in Fig 5, there is a co-localization of g7-NPs and albumin in both animal types, thus demonstrating for the first time that g7-NPs can carry high MW molecules, as albumin, across the BBB. Moreover, Fig 6 highlights a perinuclear distribution of the g7-NPs/Alb, confirming their capability to reach the brain parenchyma.

Moreover, Fig 3 shows how the number of g7-NPs/Alb per optical field is much higher in Idua-ko than in wt mice, about 4.4 fold. As control, the administration of free albumin solution (0.2 mg/mouse) in Idua-ko and wt mice (both n = 3) did not lead to any significant signals of albumin in the examined fields, thus excluding FITC-albumin BBB crossing and confirming, indirectly, the maintenance of the BBB integrity and impermeability to high MW molecules in Idua-ko mice.

As further control, we also injected untargeted NPs loaded with albumin (u-NPs/Alb) in Idua-ko (n = 3) and wt (n = 3) mice. As expected, the extent of BBB crossing is almost non-existent in both animal types (mean value: 17 and 9 u-NPs/Alb per optical field in Idua-ko and wt mice, respectively) (Figs 3 and 5), confirming that the g7-targetor moiety is needed to allow brain targeting of PLGA-NPs, as well as brain delivery of the loaded Alb.

To consider whether Alb adsorbed onto NPs (targeted or not) can be driven across the BBB, we additionally injected Idua-ko (n = 4) and wt (n = 3) mice with unloaded g7-NPs suspended in free FITC-albumin (MIX1). As shown in Fig 3 and image (a) in S2 Fig, in MIX1 FITC-albumin bound externally to the surface of the NPs does not interfere with g7 targeting ability and was able to cross the BBB. However, remarkably, Alb adsorption does not assure the stabilization, protection from degradation and the control of the release of the protein (or enzyme). Thus, even if a presence of Alb is evident within the brain parenchyma after injection of g7-NPs adsorbed Alb, we cannot propose this approach (adsorption procedure) as reliable option for enzyme replacement therapy. As supposed, the injection of unloaded u-NPs resuspended in free FITC-albumin (MIX2), did not lead to any signals of Alb in the CNS, confirming the inability of untargeted NPs to cross the BBB and bring in any adsorbed materials (as Alb) (image (b) in S2 Fig).

In conclusion, there is a higher, statistically significant, amount of g7-NPs (p-value = 0.0002) and g7-NPs/Alb (p-value = 0.0007) that can cross the BBB with respect to u-NPs and u-NPs/Alb respectively. This provides excellent prerequisites for the use of these g7-NPs with the recombinant enzymes, as well as with other high molecular weight molecules, to treat diseases affecting the neurological district.

### Study of PLGA-NPs and albumin BBB crossing in the MPS II mouse model

Efficiency of g7-NPs/Alb BBB crossing was afterwards evaluated in the MPS II mouse model (Ids-ko). These experiments aimed to extend the potential use of this kind of carriers to transport high molecular weight therapeutic molecules to the brain district in other LSDs and hopefully in many other neurological disorders.

**Fig 7:**
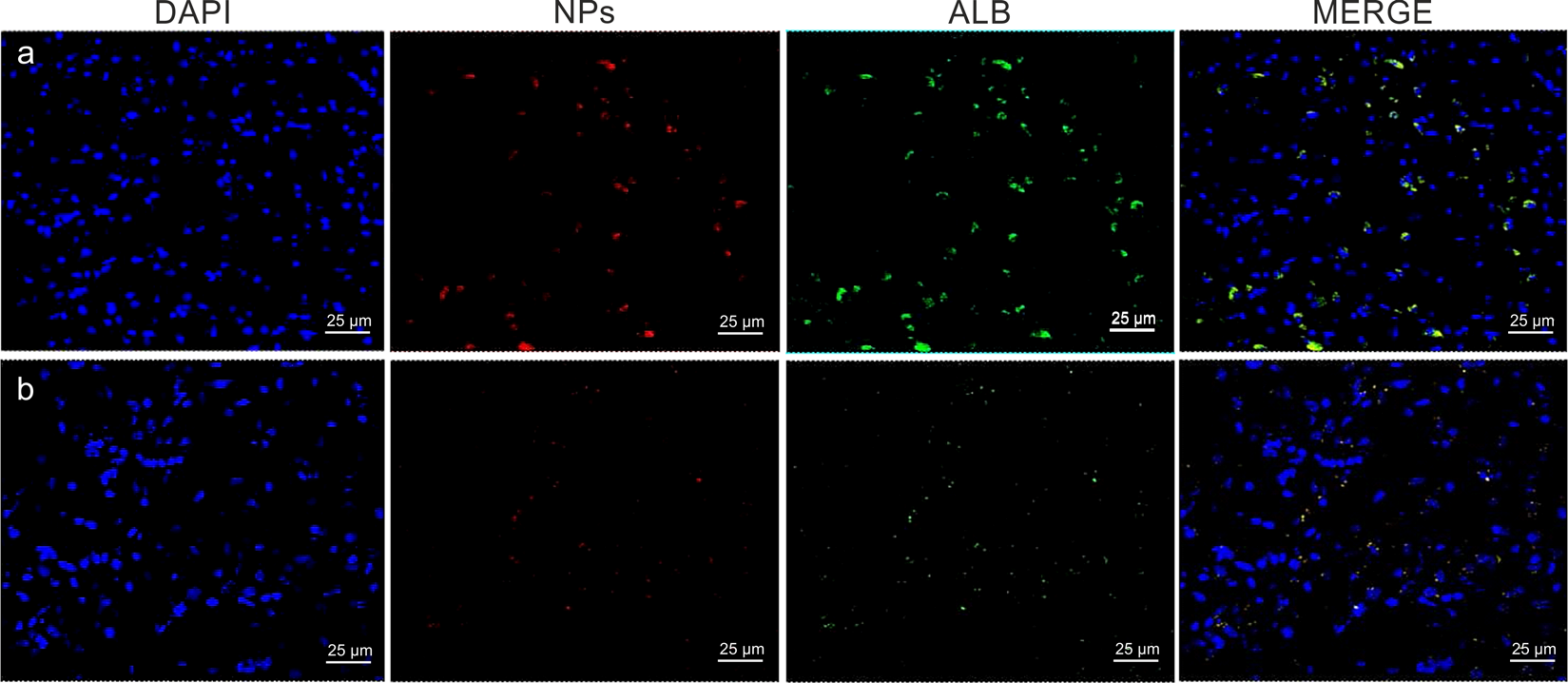
g7-NPs/Alb representative confocal images of Ids-ko and wt mice brain. (a) Ids-ko and (b) syngeneic wt mice. Nuclei are shown in blue (DAPI staining), NPs in red (rhodamine labeling), Albumin in green (FITC labeling); last column represents the merged images.

**Fig 8:**
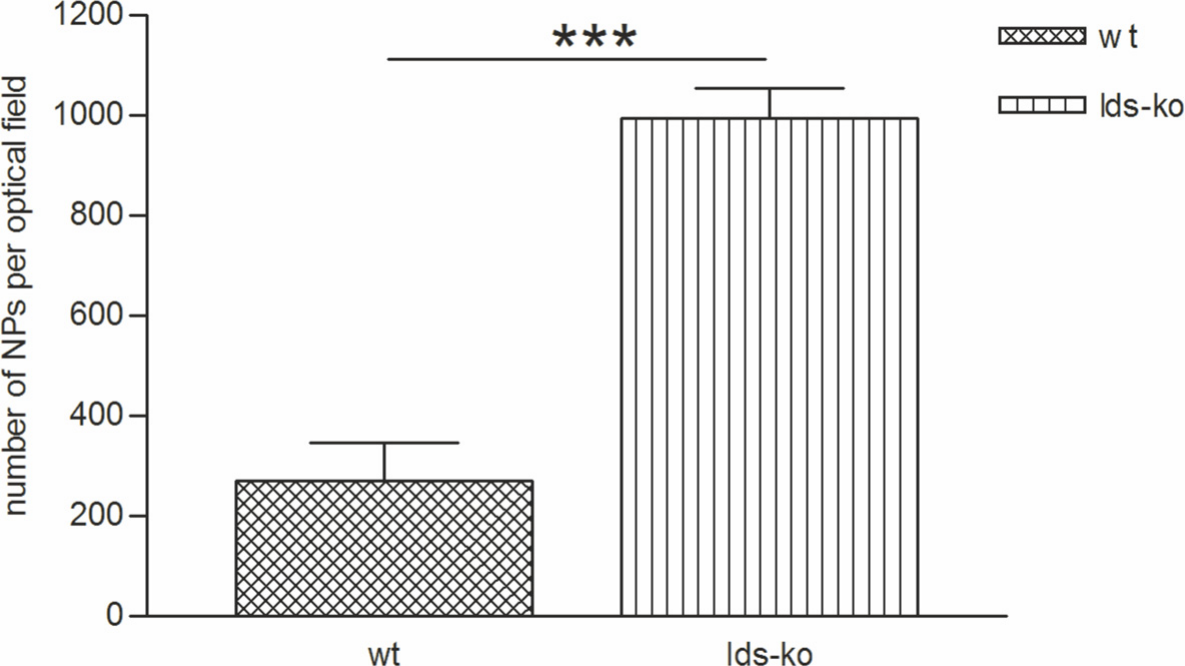
Number of g7-NPs/Alb in the brain of Ids-ko and wt mice. *p-value <0.001.

As show in Fig 7, g7-NPs/Alb can cross BBB also in the Ids-ko mouse model and, as already seen in the Idua-ko mice, we found a significantly higher (p-value = 1.52E-07) number of NPs per optical field in the Ids-ko vs the syngeneic wt mice (n = 5), about 3.6 fold (Fig 8).

In this case, the extent of g7-NPs/Alb reaching the brain is significantly lower (1.5 fold, p = 0.006) with respect to Idua-ko mice and this might be due to a different BBB permeability or modified processes in BBB crossing pathways in the two different pathologies. This finding represents an open question related to the different status of the BBB in very similar pathologies (MPS I, MPS II and MPS III), but with different outputs in terms of BBB crossing and endothelial dynamic damage.

As a control, we also injected both free albumin and Evans Blue in Ids-ko and wt mice (n = 3) and the results confirmed that they cannot cross the BBB in both types of mice (S1 Text).

## Conclusion

Clinical efficacy of conventional therapies for neurological diseases is in most cases limited by the ability of the drugs to reach the cerebral district. In addition, the rapid removal of most drugs from the bloodstream decreases their bioavailability and reduces the amount usable by the target site.

Recent studies show that carriers as the engineered nanoparticles may cross the BBB without damage and carry drugs and genetic material to the brain, thus being considered as potential strategies for the treatment of brain disorders [14–17].

Therefore the use of nanotechnologies, able to protect their content and to carry therapeutics to the CNS, may fit perfectly with the need to carry specifically to this district the recombinant enzymes necessary to correct the neurological impairment characteristic of many LSDs.

To this aim, preliminary experiments were conducted by i.v.-injecting Idua-ko, Ids-ko and wt mice with PLGA-NPs labeled with Rhodamine, modified with g7 and loaded with FITC-albumin. Results have demonstrated that the g7-NPs are able to cross the BBB and to widely localize in all brain parenchyma both in ko and wt mice, with higher efficiency in ko animals. This demonstrates the applicability of NPs for delivery of high molecular weight molecules in these two mouse models, thus encouraging their potential application to enzyme delivery to the brain.

Since in most LSDs the primary cause of neurodegeneration is the deficit of a specific enzyme, the administration of the functional protein may be sufficient to restore brain functions and help to understand at what extent such a progressive neurodegenerative process may be reversible. Moreover, the features of g7-NPs, as internalization and up-take by neurons through the clathrin pathway, submission to an intracellular trafficking process based on Rab5 and accumulation within lysosomes, represent a significant advantage since the corrective enzyme is transported to the organelle where the pathological storage is physiologically metabolized [14,15,19].

## Supporting Information

S1 Fig u-NPs and g7-NPs representative confocal images of Idua-ko and syngeneic wt mice brain. a) Idua-ko injected with g7-NPs; b) wt injected with g7-NPs; c) Idua-ko injected with u-NPs; d) wt injected with u-NPs. *Abbreviations*: Idua-ko, α-L-iduronidase knock-out mice; wt, wild-type mice; PLGA-NPs, poly-lactide-co-glycolide nanoparticles; g7-NPs, unloaded and targeted nanoparticles; u-NPs, unloaded and untargeted nanoparticles. Representative confocal images of the brain of Idua-ko and wt mice injected with targeted and untargeted unloaded PLGA-NPs.
(TIFF)

S2 Fig MIX1 and MIX2 representative confocal images of Idua-ko and syngeneic wt mice brain. Brains of the Idua-ko (a, c) and wt mice (b, d) injected with: a, b) MIX1 (g7-NPs+Alb), c, d) MIX2 (u-NPs+Alb). MIX1 (g7-NPs+Alb): unloaded targeted nanoparticles resuspended in FITC-albumin solution; MIX2 (u-NPs+Alb): unloaded and untargeted nanoparticles resuspended in FITC-albumin solution.
(TIFF)

S1 Text BBB integrity. (DOCX)
