# Supplementary figures and images for: Targeted Polymeric Nanoparticles for Brain Delivery of High Molecular Weight Molecules in Lysosomal Storage Disorders
Source: PLoS One. 2016 May 26;11(5):e0156452. doi: 10.1371/journal.pone.0156452 (PMC4881964; doi:10.1371/journal.pone.0156452)

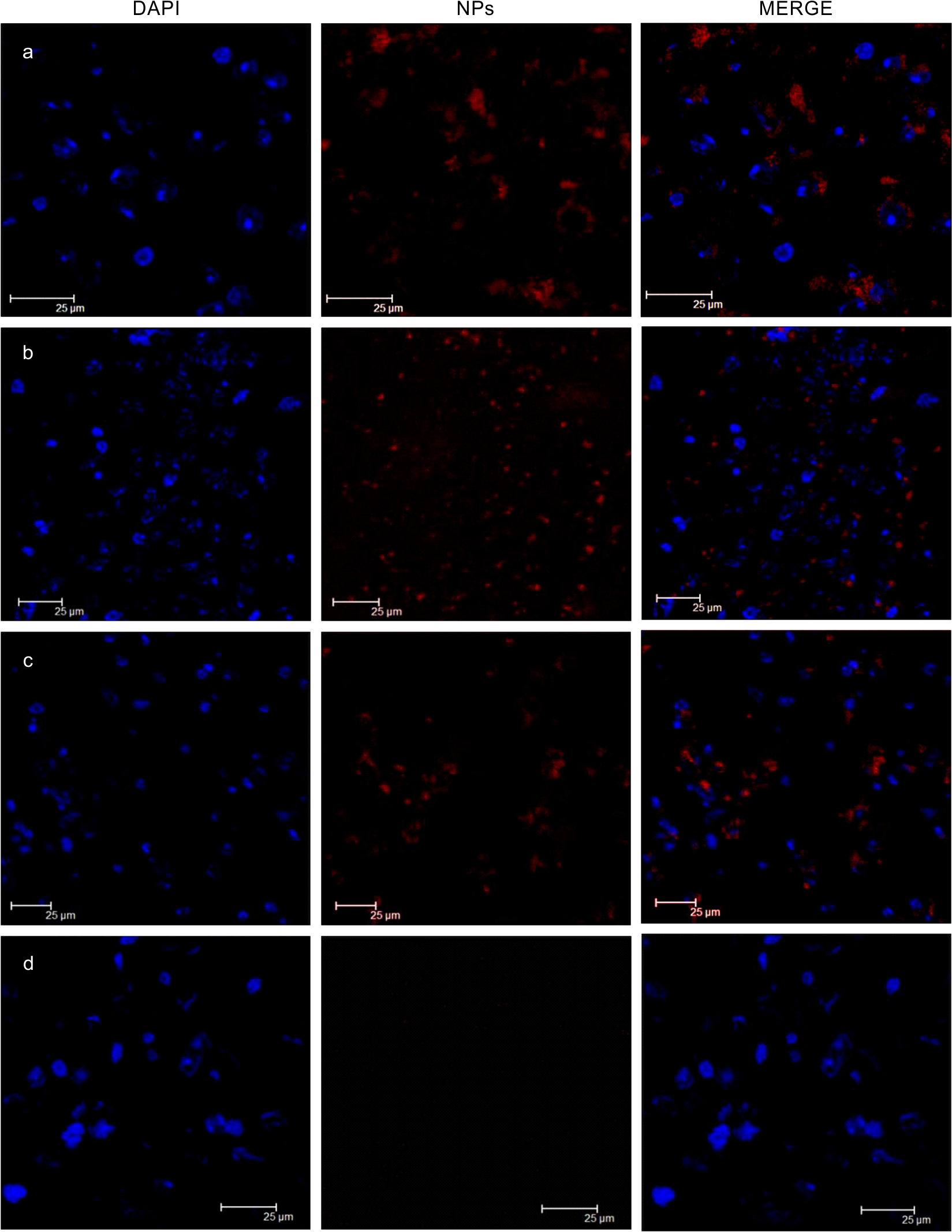

Supplement: S1 Fig — a) Idua-ko injected with g7-NPs; b) wt injected with g7-NPs; c) Idua-ko injected with u-NPs; d) wt injected with u-NPs. Abbreviations: Idua-ko, α-L-iduronidase knock-out mice; wt, wild-type mice; PLGA-NPs, poly-lactide-co-glycolide nanoparticles; g7-NPs, unloaded and targeted nanoparticles; u-NPs, unloaded and untargeted nanoparticles. Representative confocal images of the brain of Idua-ko and wt mice injected with targeted and untargeted unloaded PLGA-NPs. (TIFF) [file pone.0156452.s001.tiff]

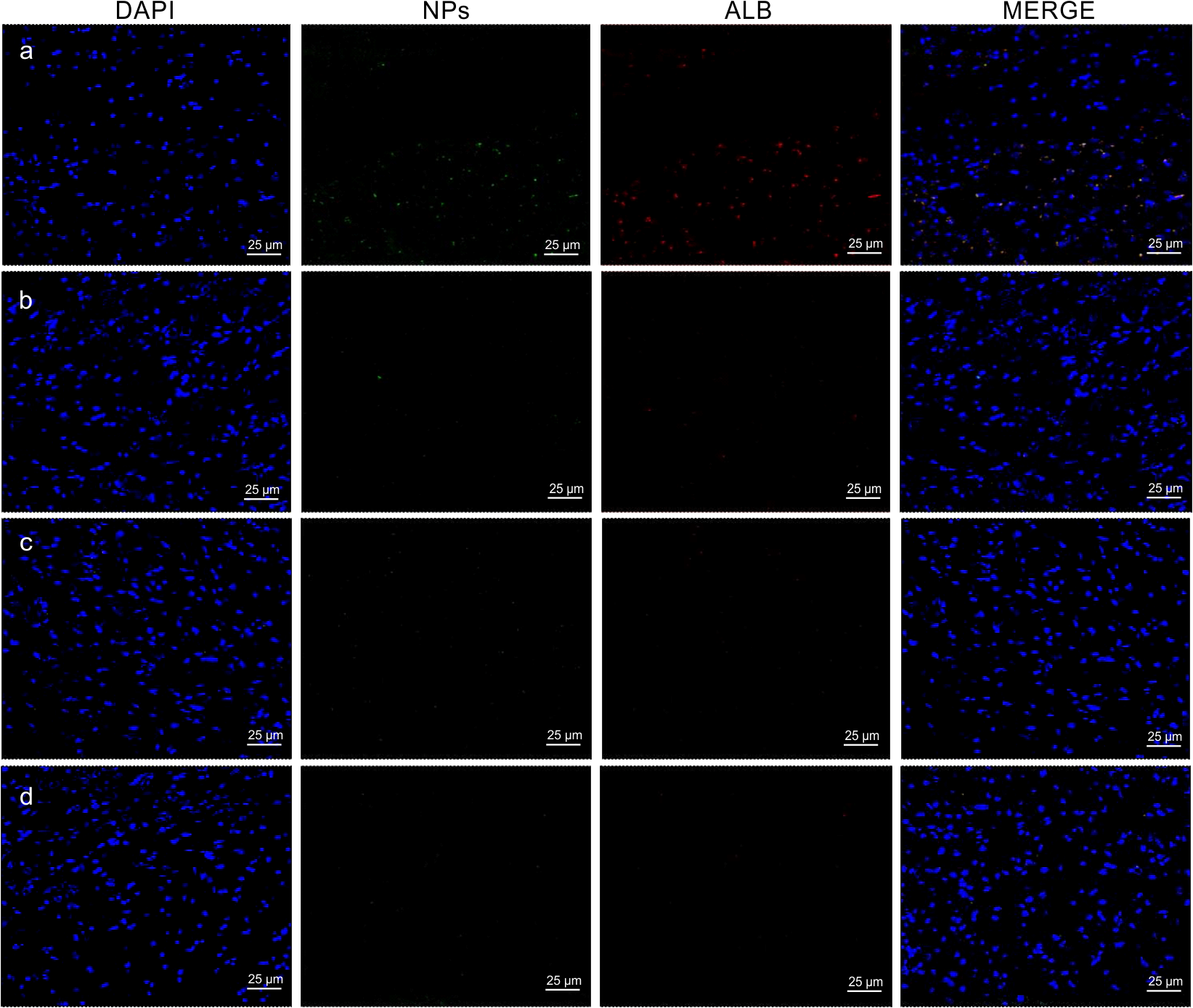

Supplement: S2 Fig — Brains of the Idua-ko (a, c) and wt mice (b, d) injected with: a, b) MIX1 (g7-NPs+Alb), c, d) MIX2 (u-NPs+Alb). MIX1 (g7-NPs+Alb): unloaded targeted nanoparticles resuspended in FITC-albumin solution; MIX2 (u-NPs+Alb): unloaded and untargeted nanoparticles resuspended in FITC-albumin solution. (TIFF) [file pone.0156452.s002.tiff]
