# Supplementary material for: Targeted Polymeric Nanoparticles for Brain Delivery of High Molecular Weight Molecules in Lysosomal Storage Disorders
Source: PLoS One. 2016 May 26;11(5):e0156452. doi: 10.1371/journal.pone.0156452 (PMC4881964; doi:10.1371/journal.pone.0156452)
Supplement: S1 Text — (DOCX) [file pone.0156452.s003.docx]

**Methods**

**BBB Integrity**

To check BBB integrity and exclude possible damages, we used Evans Blue (EB) fluorescent dye (MW: 960.8, Sigma-Aldrich), as a tracer. Since serum albumin cannot cross the barrier, and virtually all EB is bound to albumin, if BBB is intact, the neural tissue remains unstained; instead, when BBB is compromised, the albumin-bound EB enters the CNS. In this study 50 μl of a 2% solution EB in 0.9% NaCl were i.v.-injected in Idua-ko, Ids-ko and in the respective wt mice [1]. Four hours later mice were sacrificed by cervical dislocation and the brains collected and processed as described in the manuscript “Materials and methods” section.

**Results and discussion**

**Checking BBB integrity**

To exclude that the higher g7-NPs/Alb uptake in the ko mouse models may be due to possible damages in their BBB, we performed experiments aimed to check BBB integrity.

We injected Idua-ko, Ids-ko and the respective wt mice with Evans Blue solution; none of the animals showed BBB crossing of the dye, thus indicating an undamaged BBB [2]. So, the difference in the NPs uptake may hypothetically reside in other factors, as a different expression of transporters on the endothelium as well as a modification in the endocytosis process in both ko mice vs syngeneic wt controls. As previously reported in the manuscript, also the results obtained in the liver support this hypothesis.

Therefore, more studies will be necessary to understand what leads to this difference in the uptake of NPs, but most of all what are the basic differences in the BBB of both ko vs related wt mice.

**References**

1. Deng SX, Panahian N, James H, Gelbard HA, Federoff HJ, Dewhurst S, et al. Luciferase: a sensitive and quantitative probe for blood-brain barrier disruption. *J Neurosci Methods* 1998;2:159-164.

2. Kozler P, Pokorny J. Altered blood-brain barrier permeability and its effect on the distribution of Evans blue and sodium fluorescein in the rat brain applied by intracarotid injection. *Physiol Res* 2003;5:607-614.
